# Targeting DNA Damage Repair to Enhance Antitumor Immunity in Radiotherapy: Mechanisms and Opportunities

**DOI:** 10.3390/ijms26083743

**Published:** 2025-04-16

**Authors:** Lin Yang, Wenjie Wei, Xun Yuan, Ergang Guo, Ping Peng, Jing Wang, Wei Sun

**Affiliations:** Department of Oncology, Tongji Hospital, Tongji Medical College, Huazhong University of Science and Technology, Wuhan 430030, China; linyang@tjh.tjmu.edu.cn (L.Y.); cindyweiwj@163.com (W.W.); yuanxun@tjh.tjmu.edu.cn (X.Y.); ergangguo@163.com (E.G.); pengpingtjh@163.com (P.P.); jing234wang@tjh.tjmu.edu.cn (J.W.)

**Keywords:** DNA damage repair, immunotherapy, radiotherapy, targeted therapy, immune checkpoint inhibitors

## Abstract

Radiotherapy is a standard cancer treatment that involves the induction of DNA damage. DNA damage repair (DDR) pathways maintain genomic integrity and make tumors resistant to radiotherapy and certain chemotherapies. In turn, DDR dysfunction results in cumulative DNA damage, leading to increased sensitivity for antitumor treatment. Moreover, radiotherapy has been shown to trigger antitumor immunity. Currently, immunotherapy has become a new and widely used standard strategy for treating a broad spectrum of tumor types. Notably, recent studies have demonstrated that DDR pathways play important roles in driving the response to immunotherapy. Herein, we review and discuss how DDR affects antitumor immunity induced by radiotherapy. Furthermore, we summarize the development of strategies for combining DDR inhibitors with radiotherapy and/or immunotherapy to enhance their efficacy against cancers.

## 1. Introduction

Radiation therapy (RT) is an important treatment for many malignancies. RT can cause several types of DNA damage, such as basic sites, single-strand breaks (SSBs) and double-strand breaks (DSBs), and then eliminate tumor cells [1]. However, DNA damage can activate cellular DNA damage repair (DDR) signaling pathways and confer tumor cell survival and resistance to radiation [2]. Currently, targeting DDR pathways has emerged as an appealing strategy for overcoming tumor radiation resistance [3].

Typically, RT is used to eliminate cancer cells at the site that is directly radiated. However, in some cases, RT can induce a systemic anticancer response at distant tumor sites that are not subjected to RT, which is known as the “abscopal effect” [4]. A possible explanation for this interesting phenomenon is that RT can trigger antitumor immunity, which plays important roles against tumors outside the radiation field [5]. Nevertheless, the abscopal effect rarely occurs. Elucidating how to elicit more abscopal effects to control both primary and metastatic cancer sites is challenging. Thus, further exploration of the mechanisms by which RT regulates the antitumor immune response and identification of the pivotal signaling pathways or players involved in the processing are especially important.

DDR pathways are crucial for transmitting genetic material accurately [6,7]. However, various factors affect genomic integrity. DDR failure leads to increased DNA damage and high sensitivity to cancer treatment [8]. The combination of inhibitors of DDR and other antitumor methods, such as RT, chemotherapy and immune therapy, appears promising [9]. Several targets emerging from DDR pathways have been exploited for radiosensitization [10,11]. In addition, recent studies have demonstrated that cancers with DDR dysfunctions might have high tumor mutation burden (TMB) and abundant neoantigens that can trigger the immune system against tumors [12]. Synthetic lethality (SL) is a genetic interaction where the loss of function in either of two genes alone does not cause cell death, but the simultaneous loss of both genes is lethal [13]. DNA repair inhibitors can potentially exploit this concept. Tumors often contain mutations in one or more DNA repair pathways, which makes them dependent on other functioning pathways. By inhibiting a key alternative pathway, there can be a harmful accumulation of unrepaired DNA damage that ultimately leads to cell death. By contrast, normal cells have an unbroken pathway to repair this damage, allowing for selective targeting of cancer cells. Thus, inducing SL could be a strategy to overcome the limitations of cancer therapy by targeting a compensatory pathway. Considering the concept of SL, DDR inhibitors combined with DNA-damaging therapies, such as radiotherapy, chemotherapy, and immune checkpoint inhibitors, can improve the therapeutic effectiveness of cancer cells activated by the DDR pathways [14].

Currently, immunotherapy, especially immune checkpoint inhibitors (ICIs), is recognized as a landmark of cancer treatment. Although immunotherapy has improved survival and tumor control, the response to immunotherapy is still limited or short in many cancer patients [12]. Therefore, increasing the response to immunotherapy is urgently needed. Since RT can activate a systematic immune response, studies have reported that RT can synergize with immunotherapy to improve antitumor efficacy [15,16]. The combination strategy is supported by the results of the PACIFIC clinical trial, which assessed the effect of immunotherapy after definitive chemoradiation [17]. In this trial, unresectable stage III non-small cell lung cancer (NSCLC) patients who received durvalumab (an anti-programmed death-ligand 1 (PD-L1) antibody) following concurrent platinum-based chemoradiation demonstrated significantly improved progression-free survival (PFS: 16.8 vs. 5.6 months; hazard ratio [HR]: 0.52) and overall survival (OS: 47.5 vs. 29.1 months; HR: 0.72) compared to placebo. These results led to the approval of durvalumab as a standard treatment consolidation therapy. Furthermore, targeting DDR has also been demonstrated to be valuable in improving the efficacy of immunotherapy.

In view of the relationships among RT, the DDR and immunotherapy, we describe the mechanisms underlying how the DDR affects antitumor immunity during radiotherapy. We also summarize the progress of potential strategies for combining DDR inhibitors with radiotherapy to increase the efficacy of immunotherapy in the present review.

## 2. DNA Damage Repair in Radiotherapy

More than 70% of patients undergo RT during their antitumor period [18]. RT is an essential treatment for cancers that uses radiation to damage and kill cancer cells. However, tumor cells often exhibit radiation resistance, which causes RT failure. Induction of DNA damage is the main mechanism underlying the effects of radiation against cancer cells. DNA damage leads to genomic instability, cell apoptosis, changes in cell cycle checkpoint and death after mitosis.

### 2.1. DNA Damage in Radiotherapy

The major DNA lesions induced by ionizing radiation are SSBs and DSBs. After radiation exposure, SSBs arise more frequently, but DSBs cause more fatal injuries to cancer cells [19]. Moreover, a protective DDR can also be activated by ionizing radiation in human cancer cells [20,21,22]. Thus, the balance between DNA damage and the DDR system determines the sensitivity of cancers to RT. Exploring the specific process of DDR post IR would help to overcome radioresistance.

### 2.2. DNA Damage Sensors and Early Responders

The mammalian cell response to DSBs comprises a cascade of proteins that are classified as sensors, signal conductors, mediators and effectors for injury and repair [23]. During the DSB response, the MRE11-NBS1-RAD50 (MRN) complex plays an important role in signal interruption and promoting lesion repair [24]. The MRN complex functions in early DSB reactions mainly by binding DNA termini and recruiting ataxia-telangiectasia-mutated (ATM), resulting in the generation of signalling complexes comprising damaged DNA and ATM [25,26]. The protein subsets phosphorylated by ATM are subsequently redistributed into DSB-containing subnuclear compartments, including histone H2AX (H2AX) [19]. H2AX is rapidly phosphorylated after radiation, and there is a specific temporal and spatial relationship between the number of γ-H2AX lesions and DNA damage [27]. Most DSB-responsive proteins are activated by the participation of phosphoinositide 3-kinase (PI3-K)—like kinase proteins, including ATM, DNA-dependent protein kinase catalytic subunit proteins (DNA-PKcs) and ataxia telangiectasia and rad3-related (ATR) [19,28,29]. These DNA damage-sensing proteins typically respond to radiation within seconds to minutes. Highly coordinated DNA damage-sensing mechanisms ensure signal transmission between upstream and downstream proteins and maintain genomic integrity and chromosome stability in radiation-damaged cells. In turn, disabling DSB repair factors leads to genomic instability, which enables tumor development, but they also create vulnerabilities that can be used for cancer therapy. Radiosensitization, induced by inhibiting ATM, ATR or DNA-PKcs, has been demonstrated in many cancer types [3,10,11]. The development of agents that block DSB repair and can be used in combination with radiotherapy in the clinic is warranted for the treatment of cancers.

### 2.3. DNA Damage Repair Pathways

The typical DDR system includes several pathways, including the direct repair (DR) pathway, the mismatch repair (MMR) pathway, the base excision repair (BER) pathway, the nucleotide excision repair (NER) pathway, the homologous recombination (HR) pathway and the nonhomologous end joining (NHEJ) pathway [2,30]. The main mechanisms of DSB repair include HR and NHEJ. HR uses the complete sister chromatids as repair templates, and NHEJ directly connects the broken ends without templates [31]. Therefore, HR is more accurate for ensuring genomic stability. However, it only occurs during the cell cycle stages S and G2 because it relies on sister chromatids as templates for repair [32,33,34]. Thus, NHEJ is the main DDR repair pathway [35]. ATM kinases are recruited to trigger the DSB signaling cascade and coordinate the DNA repair process when the MRN complex is activated. It has been reported that ATM mutations can significantly damage the HR and NHEJ pathways, which are vital for repairing DNA damage directly caused by IR [36,37].

### 2.4. Cellular Response to Radiotherapy

In the cellular response to radiation, DNA damage sensors, such as MRN, ATM, ATR and DNA-PKcs, detect the DNA damage and trigger signal transduction pathways. p53 and nuclear factor κB (NF-κB) are the key downstream transcription factors of DNA damage recognition factors. The activation of p53 and NF-κB results in the altered expression of a series of target genes that cause cell death or survival. p53 and NF-κB both transcribe pro-survival and pro-apoptosis genes. They initially protect cells against DNA damage, but if the damage is not repaired, they become pro-apoptotic. The dual function of p53 and NF-κB supports the paradigm that low levels of DNA damage trigger their repair function, whereas high levels of DNA damage activate apoptosis [38].

## 3. Immune Response in Radiotherapy

The immune system can identify and clear malignant cells through immune editing. However, some tumors can escape the killing effects of the immune system. RT causes DNA damage, especially DSBs, ultimately leading to cell death. During this process, RT has the potential to trigger an antineoplastic immune response via the production of fragmented DNA and the release of tumor neoantigens, which can increase the invasion of lymphocytes into the tumor matrix and change the tumor immune microenvironment [5,39,40]. In this situation, radiation therapy may act as an in situ inoculation, which leads to systematic immunological recognition and regression of whole-body lesions, as evidenced by the “abscopal effect” [41].

The activation of the immune response by RT suggests the combinational effect of RT and immunotherapy. Currently, the result of the PACIFIC clinical trial is the most established clinical evidence for the combination. An important challenge is determining the optimal dose and fractionation of radiation for the combination. Recent evidence has demonstrated that, compared with conventionally fractionated RT, hypofractionated RT or stereotactic radiation body therapy (SBRT) may improve the synergistic effects of RT and immunotherapy because it is less detrimental to immune cells and results in the increased production of cytosolic DNA and tumor neoantigens [42,43].

### 3.1. Radiation and Innate Immune Signaling

**Figure 1:**
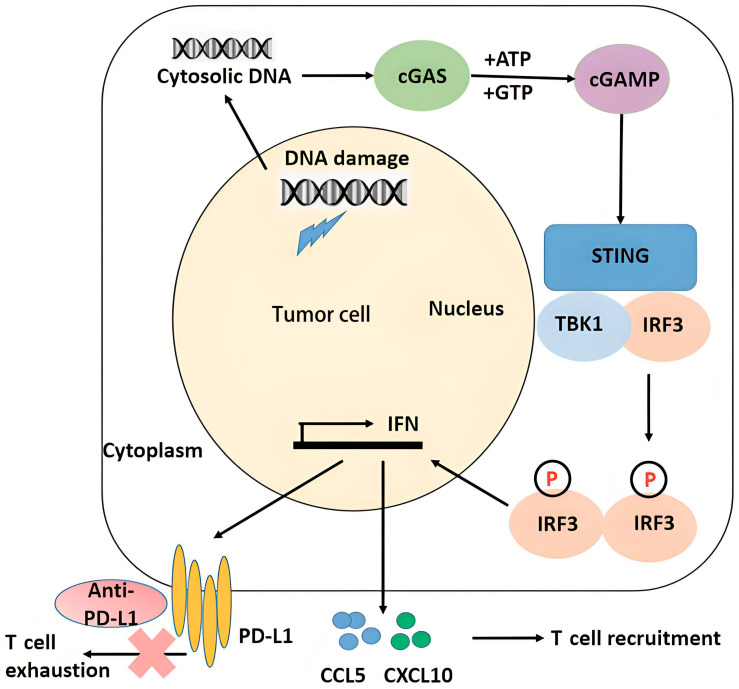
Radiation induces tumor cytosolic DNA and triggers innate immune responses through the cGAS-STING pathway. cGAS, cyclic GMP-AMP synthase; STING, stimulator of interferon genes; ATP, adenosine triphosphate; GTP, guanosine triphosphate; TBK1, ANK binding kinase 1; IRF-3, interferon regulatory factor 3; IFN, interferon; CCL, C–C motif chemokine ligand; CXCL, C–X–C motif chemokine ligand; PD-L1, programmed death-ligand 1; the red X sign means “blocking”.

Radiation damages DNA in tumor cells directly or indirectly through free radicals. Damaged DNA, which is fragmented DNA, translocates from the nucleus to the cytoplasm, and is called cytoplasmic DNA. Cytoplasmic DNA can be detected and trigger innate immune responses. Cyclic GMP-AMP synthase (cGAS) is the most crucial DNA sensor. Once cGAS recognizes cytoplasmic DNA, it activates the cGAS-stimulator of interferon genes (STING) pathway, subsequently leading to the phosphorylation of interferon regulatory factor 3 (IRF-3) and TANK binding kinase 1 (TBK1). Phosphorylated IRF-3 and TBK1 then increase the production of type I interferons (IFNs), which, together with other chemokines, activate the aggregation of immune cells at tumor sites [44,45,46,47]. Meanwhile, IFNs upregulate the expression of PD-L1 in tumor cells, forming an immunosuppressive feedback loop, but at the same time providing a synergistic therapeutic target for radiotherapy combined with anti-PD-L1 immune checkpoint inhibitors. T cell exhaustion is a state of dysfunctional T cell activity characterized by upregulated inhibitory receptors (such as PD-1) and impaired cytokine production [48]. Radiotherapy-induced DNA damage promotes PD-L1 upregulation on tumor cells, which engages PD-1 on T cells to drive exhaustion [49]. Anti-PD-L1 agents block this interaction, restoring T cell effector functions and reversing exhaustion (Figure 1). For example, in the PACIFIC trial, consolidative durvalumab after chemoradiotherapy significantly prolonged survival in NSCLC patients, demonstrating that PD-L1 blockade mitigates radiotherapy-associated T cell exhaustion. In addition, Melanoma 2 (AIM2) is another important cytoplasmic DNA sensor. It is reported that AIM2 promotes irradiation resistance, migration ability and PD-L1 expression [50]. Moreover, the absence of AIM2 leads to inflammasome assembly, which causes the release of proinflammatory factors and accelerates cell death [51,52].

### 3.2. Radiation and Adaptive Immune Signaling

Innate and adaptive immunity cooperate together to produce a systemic immune response. In addition to innate immunity, radiation can also induce adaptive immunity [53,54,55,56]. Radiation causes tumor cell death, after which antigen-presenting cells (APCs) phagocytose these cells and submit antigens to T cells to trigger an adaptive immune response. Moreover, RT facilitates the translocation of calreticulin (CRT) from the endoplasmic reticulum to the plasma membrane [57] and downregulates proteins that induce the antiphagocytic signal CD47 [58]. Surface-exposed CRT acts as an “eat-me” signal, promoting phagocytosis of dying tumor cells by dendritic cells (DCs) and macrophages [57]. The increasing trend of radiation-induced CRT exposure on the surface of melanoma cells was related to the enhanced phagocytic activity of DCs on irradiated melanoma cells [59]. This process enhances antigen cross-presentation and subsequent T cell priming.

Radiation modulates the immune response in other ways, such as eliciting signals that stimulate toll-like receptor-4 (TLR-4) on DCs [54]. Dead cells emit danger signals, called damage-related molecular patterns, which promote the maturation and activation of DCs. Then DCs phagocytose tumor cells and present their antigens to T cells via major histocompatibility complex (MHC) molecules, which stimulate the initiation and activation of T cells.

### 3.3. Radiotherapy and Abscopal Effects

**Figure 2:**
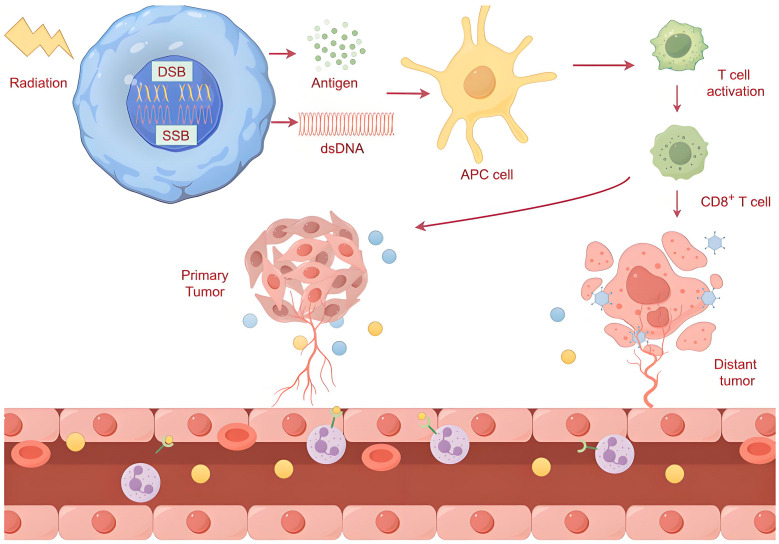
Radiation activates antitumor immune responses both in the primary and at distant cancer sites. DSB, double-strand break; SSB, single-strand break; dsDNA, double-stranded DNA; APC, antigen-presenting cell.

When cancer patients receive local radiation, antitumor responses are observed in tumors far from the irradiated site, which is called the abscopal effect. The abscopal effect provides evidence for the immune activation of RT, suggesting that RT not only damages tumors directly but also triggers antitumor immunity throughout the whole body. This immune response liberates different chemokines so they can enter the tumor environment, causing DCs to be recruited to the site of the tumor [60]. For instance, cytokines including interleukin (IL)-6, IL-1α and TNF-α were significantly elevated after irradiation, which was accompanied by macrophage activation [61]. A clinical trial with lung cancer patients showed that elevated post-radiation IFN-β levels correlated with improved abscopal responses and survival [62]. DC activation and T cell upregulation may be the mechanisms underlying the abscopal effect [63,64]. When radiation is applied to tumor cells, the cellular stress or DNA damage causes the release of neoantigens. Neoantigens are phagocytosed by APCs and presented to cytotoxic T cells, which can identify and attack tumors both in the primary site and at distant sites (Figure 2). In addition, transforming growth factor-β and other immune factors can be released into the extracellular chamber and activate the antitumor immune response [65]. High radiation doses in wild-type p53 tumors induce the onset of a senescence-associated secretory phenotype and the secretion of CD63^+^ extracellular vesicles that convey senescence messages outside of the irradiation field, triggering the abscopal effect in non-irradiated tumors [66].

Considering the abscopal effect, multiple immune-targeting strategies in combination with radiotherapy are currently being investigated in various cancers. These strategies include the combinational administration of cytokines, the transfer of immune cells with antitumor activity, gene-mediated cytotoxic immune therapy, vaccine therapy, immune checkpoint inhibitors, co-stimulatory agonists, and immunotherapy targeting the tumor microenvironment [65].

## 4. DDR and Immunotherapy

Currently, immunotherapy has become an important therapy for several types of cancers. To improve the limited response rate of immunotherapy, biomarker selection in the patients who are likely to respond to immunotherapy and the exploration of suitable combinations are two current research focuses. DNA damage can induce genomic instability and elicit immune responses. DDR dysfunction potentiates this process, suggesting that the DDR is closely correlated with immunotherapy.

### 4.1. Predictive Function of the DDR in Immunotherapy

The mature immunotherapy predictors include PD-L1 expression, DNA mismatch repair (MMR) deficiency and the tumor mutation burden (TMB). However, neither of these methods is completely reliable for selecting cancer patients for immunotherapy. In addition, the cutoff values of PD-L1 expression and TMB for predicting immunotherapy response remain unclear [67]. As the DDR affects the communication between cancer cells and the host immune system, DDR alterations could act as reliable predictors for the clinical screening of immunotherapy [15].

Proteins identified in the DDR pathways can be divided into five major groups, including MMR, NER, BER, HR and NHEJ. MMR defects are the best predictive biomarkers for immunotherapy [68]. The main MMR genes included MSH2, MSH6, MLH1 and PMS2. Deficient expression of any of these genes results in a molecular signature of microsatellite instability-high (MSI-H) conditions [69]. Cancers with an MSI-H phenotype have a high TMB and neoantigen burden, indicating a significant response to ICIs. Owing to the good response of MSI-H cancers to ICIs, the anti-PD-1 drugs nivolumab and pembrolizumab are used in advanced MMR-defective or MSI-H solid tumors [70,71].

HR is a crucial pathway for accurate DSB repair and plays an essential role in maintaining genomic stability. Several studies have shown that HR defects are associated with high antigen loads, increased PD-L1 expression and prominent immune cell infiltration [72,73]. BRCA2, a core member of HR, is overexpressed in anti-PD-1 responders with melanoma. The exonuclease domains of major nuclear polymerases are encoded by DNA polymerase genes e (*POLE*) and d (*POLD1*). *POLD1*- and *POLE*-mutated tumors had significantly greater point mutation burdens, increased TIL counts and increased PD-1/PD-L1 expression, and ICI therapy has strong and lasting benefits [74,75].

In total, over 450 proteins have been reported to be correlated with the DDR pathways. Changes in a single gene may have a limited impact on the overall function of the DDR. Thus, studying the status of DDR genes allows for a more complete understanding of the overall capabilities of the DDR and a more accurate prediction of the response to ICIs. Some researchers have focused on 34 DDR genes in metastatic urothelial carcinoma patients receiving atezolizumab (an anti-PD-L1 monoclonal antibody) or nivolumab. They reported that the presence of harmful DDR alterations was related to high response rates and survival times [76].

### 4.2. Targeting the DDR Enhances the Effect of Immunotherapy

Immunotherapy is approved for a broad range of tumor types, but the durable response rate of immunotherapy ranges from 10% to 20%. Thus, combination strategies need to be explored to improve the clinical outcomes of immunotherapy. Many studies have revealed that immunotherapy combined with chemotherapy or radiotherapy has a significant survival benefit. For example, according to the results of the PACIFIC study, durvalumab is approved for the standard maintenance treatment of locally advanced NSCLC after concurrent chemoradiotherapy [77]. On the basis of the findings of the KEYNOTE189 trial, pembrolizumab in combination with chemotherapy is considered as a first-line treatment for advanced or metastatic NSCLC [78].

Apart from the predictive function of DDR pathways in immunotherapy, agents that target the DDR pathway are highly important for cancer treatment. Targeting DDR pathways may reshape the immune environment and contribute to the sensitization to immunotherapy. There are two main underlying mechanisms of this synergy. Firstly, DDR deficiency leads to the accumulation of damage to DNA and the production of neoantigens, which elicit an antitumor immune response, such as CD8^+^ T cell infiltration [79,80]. Second, damaged DNA can be transferred from the cell nucleus to the cytoplasm, activating stimulants of the interferon gene and triggering an innate immune response [81]. For example, cancer cells harboring *BRCA1/2* or *ATM* mutations exhibit a lasting response to ICIs [82]. Currently, several clinical trials are testing drugs that target the DDR pathway in combination with immunotherapy, such as cyclin-dependent kinase 4/6 (CDK4/6) inhibitors, poly (ADP-ribose) polymerase (PARP) inhibitors and ATR kinase inhibitors [67].

## 5. Targeting the DDR Enhances Antitumor Immunity in Radiotherapy

**Figure 3:**
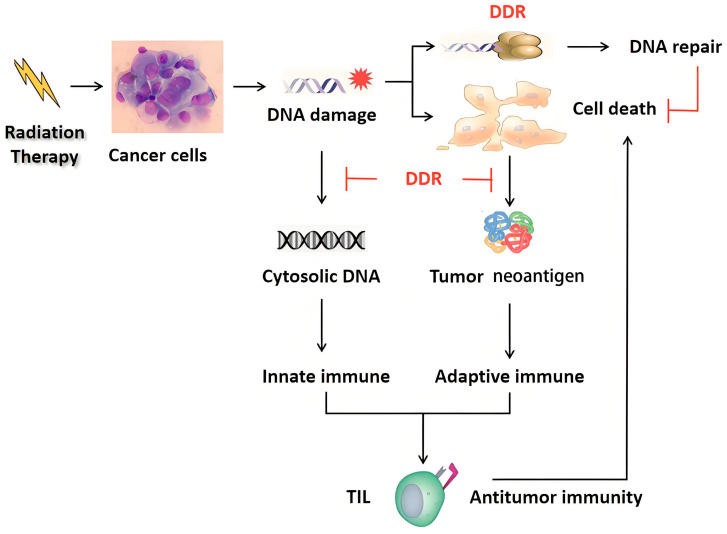
DDR of cancer cells repairs damaged DNA and causes resistance to radiotherapy and antitumor immunity. DDR, DNA damage repair; TIL, tumor infiltration lymphocyte; the black and red arrows represent induction and blocking, respectively.

Mechanistically, radiotherapy causes DNA damage, increases cytoplasmic DNA and promotes neoantigen production, which triggers the immune responses. DSBs produced by radiation are the most potent molecular events causing cancer death. However, the DDR of cancer cells can repair damaged DNA and lead to cellular radioresistance (Figure 3). Thus, targeting the DDR is a promising therapeutic method to increase the radiosensitivity of cancer cells. Moreover, targeting the DDR aggravates DNA damage, induces more cytosolic DNA and tumor neoantigens, reshapes the immune environment and then enhances antitumor immune responses, suggesting possible synergy with immunotherapy. In fact, cancer patients with DDR gene alterations do have improved response and survival rates when receiving immunotherapy.

Given the close relationship between the DDR, radiotherapy and antitumor immunity, targeting the DDR in radiotherapy not only improves the effect of radiation but also potentiates the immune response, which supports the potential of combining strategies with immunotherapy. Several important DDR-associated proteins, which are potential therapeutic targets for enhancing antitumor immunity during radiotherapy, are discussed below.

### 5.1. PARP

PARP is a vital regulatory factor of the DDR, particularly in DSB repair [83]. As DSBs are the most common type of DNA damage that causes cancer cell death during radiotherapy, PARP inhibitors may increase sensitivity to radiotherapy and enhance the immune response. Mechanistically, PARP inhibitors prevent DNA repair, increase DNA damage and induce cytoplasmic DNA and neoantigen production, which stimulates the interferon pathway and initiates antitumor immunity. PARP inhibitors have been approved to treat several solid tumors harboring deleterious germline mutations in *BRCA1/2* [84]. *BRCA1* and *BRCA2* are vital elements in the HR pathway of DSB repair. Typically, tumor cells lacking either of these proteins are sensitive to PARP inhibitors.

Niraparib, a PARP inhibitor, prevents the DDR in cancer cells and enhances the sensitivity of cancer cells to radiotherapy [85]. NCT03532880, a phase I clinical trial, is evaluating the safety of olaparib combined with radiotherapy in patients with SCLC. In addition, olaparib in combination with RT has also been studied in other tumors (NCT01562210, NCT02227082 and NCT02229656). Moreover, two phase II trials, the TOPACIO (NCT02657889) and MEDIOLA (NCT02734004) trials, reported the efficacy of PARP inhibitors in combination with ICIs in advanced solid cancers irrespective of *BRCA1/2* status [86]. PARP inhibitors combined with RT are capable of promoting the invasion of CD8^+^ T lymphocytes into the tumor bed and increasing the expression of PD-1/PD-L1. Therefore, the addition of immunotherapy with RT and PARP inhibitors synergistically improved the antitumor efficacy [87]. However, nowadays, few in vitro and in vivo studies have focused on the triple therapy that combines PARP inhibitors, radiation and ICIs, which is warranted in the future.

### 5.2. ATM/ATR

Both ATM and ATR are key mediators of the DDR. They are large polypeptides, and they have some common substrates and overlapping functions [88]. ATM is activated and recruited by the MRN complex, whereas ATR is stimulated and interacts with the ATR-interacting protein. As ATM and ATR kinases are able to promote DDR and mediate cell cycle arrest, they have the potential to improve the efficacy of radiotherapy [11]. Several ATM/ATR inhibitors have been reported to sensitize cancer cells to IR [89,90]. Furthermore, a previous study revealed that the inhibition of ATM or ATR can enhance the antitumor effect of ICIs. AZD6738, an ATR inhibitor, has been explored in combination with durvalumab (NCT03780608). The data revealed an acceptable toxicity and hopeful initial antitumor activity [91]. These results indicate that ATM/ATR may be potential targets for radiosensitization and synergy with immunotherapy. Experiments and clinical trials are needed in the future.

### 5.3. WEE1

The repair of DNA damage requires time. WEE1, a protein kinase that activates the G2/M cell cycle checkpoint, provides time for DDR [92]. The inhibition of WEE1 results in cell cycle arrest during the G2/M phase, restrained DDR and increased replication stress. Targeting WEE1 has been identified as a potential approach for radiotherapy. A previous study revealed that AZD1775, a WEE1 inhibitor, sensitizes oral tongue squamous cell carcinoma cells to radiation irrespective of TP53 status [93]. PD0166285, another reported WEE1 inhibitor, is able to increase radiosensitivity, as it prevents cancer cells from executing the DDR caused by radiotherapy [94]. Currently, a clinical trial has investigated AZD1775 in combination with durvalumab [95]. These results may indicate the possibility of combining WEEI inhibitors with immunotherapy. Further studies are warranted to assess strategies that leverage the combination of WEEI inhibitors, radiotherapy and immunotherapy.

### 5.4. CHK1

Another protein kinase required for cell cycle arrest and the activation of DNA repair is CHK1 [96]. Since CHK1 significantly influences cell survival and proliferation, CHK1 inhibition has been investigated as a potential strategy to sensitize cancer cells to radiotherapy. PF-00477736, a selective CHK1 inhibitor, was shown to contribute to radiosensitization in several cancer cell lines [97]. CCT244747, a CHK1 inhibitor administered orally, sensitizes several types of cancer cells to radiation by modulating G2/M checkpoint control [98,99]. Recently, preclinical studies have shown that inhibitors of CHK1 are able to potentiate the efficacy of ICIs in SCLC [100,101]. Further evaluation in clinical trials is needed to investigate the combination’s safety and efficacy. The combination of CHK1 inhibitors, radiotherapy and immunotherapy is also valuable for assessment in clinical trials in the future.

### 5.5. DNA-PKcs

DNA-PKcs recognize IR-induced DSBs and promote broken end management and DNA binding by recruiting proteins responsible for DDR processing and connecting the broken DNA termini [102]. To date, DNA-PKcs are the best-known regulators of the DDR. Targeting DNA-PKcs is considered an ideal therapeutic method for cancer therapy, especially for radiotherapy. In chondrosarcoma cells, the suppression of DNA-PKcs leads to sensitivity to irradiation via cell cycle arrest and telomere capping disruption [103]. Targeting DNA-PKcs is recognized as an effective method to improve the clinical outcomes of cancer patients [10]. The latest studies reported that human DNA-PKcs can drive a robust and broad immune response [104]. A clinical trial (NCT03724890) is recruiting participants to determine a safe and sustainable dose of a DNA-PKc inhibitor (nedisertib) in combination with ICI (avelumab, a PD-L1-targeted drug) with or without radiotherapy in selected patients with advanced solid tumors. Promising results are expected.

## 6. Conclusions

**Figure 4:**
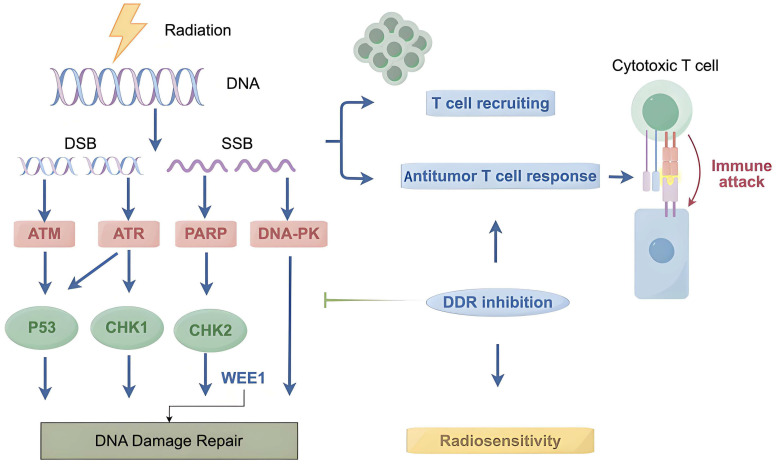
The mechanisms of combination strategies with agents targeting DDR, radiotherapy and immunotherapy. DSB, double-strand break; SSB, single-strand break; ATM, ataxia-telangiectasia-mutated; ATR, ataxia telangiectasia and rad3-related; PARP, poly (ADP-ribose) polymerase; DNA-PK, DNA-dependent protein kinase; CHK, checkpoint kinase; WEE1, Wee1-like protein kinase; the blue and black arrows represent induction, and the green represents blocking.

As discussed above, radiation causes DNA damage, which destroys the integrity of the genome. Upon exposure to IR, DNA damage signaling kinase cascades are initiated and conduct cell cycle arrest, apoptotic induction and transcriptional activation. Moreover, a series of cellular DDR pathways is triggered, including the DR, BER, MMR, NER, NHEJ and HR pathways. Radiation can potentiate innate and adaptive immunity through the induction of fragmented DNA and the release of tumor neoantigens, which can be disrupted by the DDR system. Thus, in mechanistic terms, combination strategies with agents targeting DDR, radiotherapy and immunotherapy are feasible (Figure 4). At present, preclinical and clinical studies have shown that the combination of DDR inhibitors, including blockers of PARP, ATM/ATR, WEE1, CHK1 and DNA-PKcs, with immunotherapy results in promising antitumor effects. Early-stage clinical trials assessing the triple combinations of DDR inhibitors, radiotherapy and immunotherapy are still ongoing. Future basic and clinical studies are still warranted to uncover the underlying mechanisms by which the DDR affects antitumor immunity during radiotherapy, and to further verify the efficacy of the triple combination strategy in the treatment of various types of cancer.

## Data Availability

Not applicable.
